# Case report: Morphological changes evident after manual therapy in two cases of late-diagnosed developmental dysplasia of the hip

**DOI:** 10.3389/fped.2022.1045812

**Published:** 2023-01-26

**Authors:** Christian J. Fludder, Braden G. Keil, Melissa J. Neave

**Affiliations:** ^1^ Private Practice, Melbourne, VIC, Australia; ^2^ Private Practice, Sydney, NSW, Australia

**Keywords:** developmental dysplasia of hip (DDH), manual therapies, x-Rays, growth and development, pediatrics

## Abstract

**Background:**

Late diagnosed Developmental Dysplasia of the Hip (DDH) is the detection of DDH after 3 months of age and is associated with significantly poorer outcomes than when diagnosed and managed early. Late diagnosed DDH has lower rates of success with bracing, higher rates of surgery and higher rates of complications, including avascular necrosis of the femoral head and early osteoarthritis of the hip. We describe two cases of late-diagnosed DDH which demonstrated changes in femoroacetabular joint morphology on radiographic interpretation after a 6-month trial period of manual therapy.

**Case Presentation:**

Two cases (13 and 30 months of age) with late-diagnosed DDH presented to a private chiropractic clinic for conservative, non-bracing management. One case had unilateral DDH and the other bilateral DDH. A trial of manual therapy was utilized over a 6-month period. Both cases demonstrated changes to femoroacetabular morphology as well as improvements in gross motor activity and lower extremity muscle tone.

**Conclusion:**

Manual therapy, as an adjunct or alternative to static bracing, may be of benefit in individuals with late-diagnosed DDH not responding to bracing, and prior to more invasive interventions. Additional cases of manual therapy-based management of this condition are required to inform the design of future trials to investigate this hypothesis.

## Introduction

Developmental Dysplasia of the Hip (DDH) is a common orthopaedic condition typically detected prior to 12 weeks of age at an incidence of seven per 1,000 births (1). It results in an incongruency of the femoral head relative to the acetabulum, leading to alterations in femoral head position, joint mobility and ultimately the deformational plastic changes involving the acetabulum and femoral neck which become observable on x-ray (2–6).

Hip Abduction test, Allis test and Ortolani's test are routinely utilised by medical and allied health professionals to aid in detecting this condition (1, 7). When detected prior to 3 months of age, management options depend on the severity of the dysplasia as measured by ultrasound. For those demonstrating physiological immaturity or mild dysplastic changes, observation is an appropriate management option (3, 8). For more severe dysplastic changes, dynamic bracing involving a Pavlik harness, or static bracing involving a Rhino or Denis Browne brace are commonplace (9).

Detection of DDH beyond 12 weeks of age is defined as late diagnosed DDH. There is some debate as to whether late diagnosed hips are undetected by early screening methods or have developed later in a previously stable normal developing hip, or are a combination of both. Late diagnosed DDH occurs at an incidence of approximately 0.77–1.28 per 1,000 births and appears to be increasing Australia wide (5, 6). Reasons for this increase have not been identified, however, in Australia, variability exists in hip assessment guidelines (10), and as cases of late diagnosed DDH have fewer of the traditional risk factors for DDH they may not be as closely monitored (11). Improved adherence to clinical screening recommendations and guidelines for the 3–12 months of age population may help with earlier detection of late diagnosed DDH (1). Likewise, routine ultrasound screening programs, typically performed at 6–10 weeks of age, have not consistently been shown to reduce the likelihood of late diagnosed hip dysplasia (6, 12–17). Beyond 6 months of age, x-ray is the imaging modality of choice for diagnosis and monitoring progression (3).

Early detection is critical as late diagnosed hip dysplasia often results in long term complications and disability. The likelihood of surgical interventions such as closed or open reduction, and femoral or pelvic osteotomies are much higher as are risks for avascular necrosis of the femoral head with early osteoarthritis and associated need for arthroplasty (18–20).

Treatment with dynamic bracing prior to 6 months of age is both low-risk and effective, with the success rate of the Pavlik harness in this age group being over 90% (9). Beyond 6 months of age there are limited data to support the use of harnessing, however one comparative analysis demonstrated improvements in acetabular angle in infants over a 6-month period, but bracing was unable to bring hips to a normative range (21). There is a paucity of publications or guidelines regarding utilizing bracing for children over 12 months of age with stable hip dysplasia.

A key mechanism in the development of a normal joint morphology is mechanotransduction. This is a mechanism where the cell converts mechanical stimulus into electrochemical activity. Chondrocytes sense and convert the mechanical signals into biochemical signals which direct and mediate matrix building and degrading leading to change in architecture of the joint (22–24). Appropriate mechanical forces generated by muscle tone and ambulation, when applied to the acetabular joint surfaces, direct hip joint growth and development as well as development of the femoral neck. Hip joint bracing attempts to make use of this mechanism to improve hip joint development and mechanotransduction may explain some of the changes observed as a result of manual therapy.

We report two cases of late diagnosed DDH who underwent a trial of manual therapy and demonstrated improvements in acetabular angle, femoral neck-shaft angle, and DDH severity as determined by the International Hip Dysplasia Institute (IHDI) after a three-month period with further improvement apparent after six months (25). One case had unilateral left DDH and the other case bilateral DDH. One case was closely monitored using repeat ultrasound to 14 weeks of age and was classified as normal at that age with subsequent development of DDH by 9 months of age, the second case was not diagnosed until 30 months of age. We hypothesize that the changes seen after 6 months of manual therapy treatment are the result of restoration of normative mechanotransductive activity in the hip joint, promoting changes recorded with the femoroacetabular angle. These changes are supported by a biologically plausible mechanism, however further research to support this hypothesis is required. This case report was prepared following CARE guidelines (26).

## Case 1

### Patient information

The patient was a 13-month-old female born at 37 + 6 weeks gestation in a breech presentation, weighing 2.68 kg, *via* Emergency Caesarean section at a private hospital in Australia. The mother was P1G1 and was diagnosed with anemia and low serum Pregnancy Associated Plasma Protein-A (PAPP-A) during pregnancy. There was no history of oligohydramnios nor family history of hip dysplasia. There were no difficulties with breastfeeding, nor with introduction of solids. Independent sitting was achieved at 7–8 months of age, and hand/knee crawling at 10 months of age. Independent walking had not yet been achieved; however, she was able to cruise with assistance for a short period.

The patient was assessed by a pediatrician at 4 days of age, with findings suggestive of hip subluxation but not dislocation, with normal hip abduction noted in the clinical notes. She was referred for pediatric orthopedic management after assessment by a professor at a major tertiary referral teaching hospital in Australia. An ultrasound at 31 days of age demonstrated physiological hip immaturity (Graf hip type 2a+ bilaterally) and asymmetry (Alpha angle 57° right, 50° left) with further review recommended. At this stage, bracing was not recommended as her presentation was attributed to physiological immaturity. Clinician notes at this time recorded mild laxity and normal muscle tone.

At 8 weeks of age (1M28D) repeat ultrasound demonstrated persistence of physiological immaturity present bilaterally (Graf hip type 2a+), however alpha angles were consistent with hip dysplasia (Alpha angle 57° right, 56° left). Observation without bracing was recommended and review in a further 6 weeks. Clinical notes recorded normal hip abduction and negative Barlow test.

At 14 weeks of age (3M9D), ultrasound review revealed that alpha angles had increased (72° right, 61° left), with mild changes to left acetabular superior bony rim, however both hips were classified as sonographically normal (Graf type 1b). Clinical review recorded normal abduction, with Barlow's test negative bilaterally. A recommendation was given to avoid swaddling and follow up review with her General Practitioner (GP) at 9 months of age.

**Figure 1:**
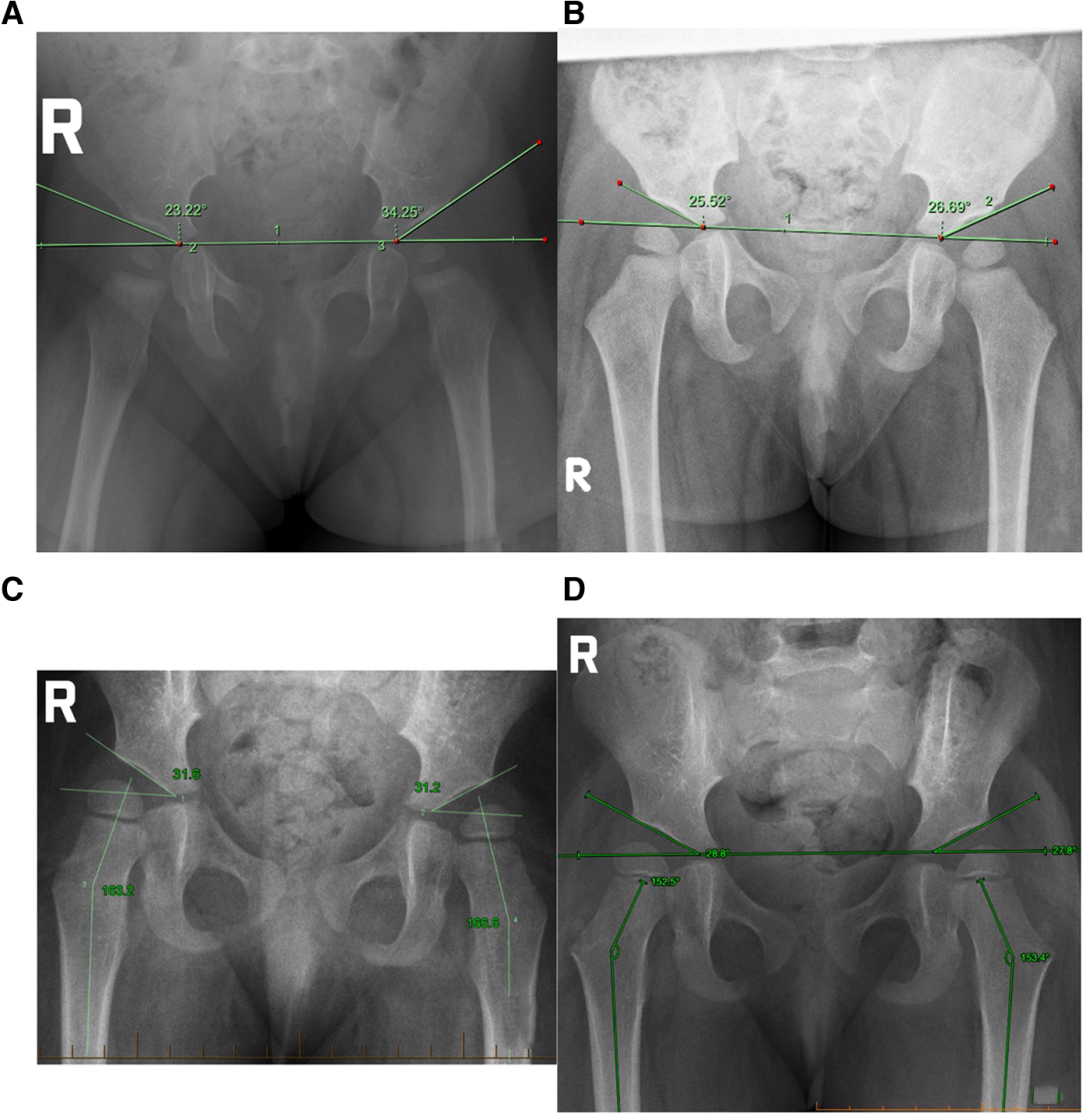
(**A**) Case 1 initial presentation at 8M29D, (**B**) case 1 after trial of manual therapy 18M30D, (**C**) case 2 at initial presentation 2Y6M3D, (**D**) case 2 after a trial of manual therapy 2Y11M10D.

The next hip review occurred at 9 months of age (8M29D) after relocation to another major Australian city, where a routine x-ray was arranged by a GP. Imaging revealed a right acetabular angle of 24°, and a left acetabular angle of 34.5°, and a clear disruption to Shenton's line signifying left acetabular dysplasia (Figure 1A).

Rhino bracing for 23 h per day for a period of 12 weeks was initiated following a private paediatric orthopaedic consultation. The parents were advised that there may be difficulty with tolerating the brace, and after one week a contact rash appeared prompting a shift to Denis Browne brace for use during naps and night-time only.

Review radiograph at 12M8D demonstrated a one-degree improvement in left acetabular angle to 33°. A further 12 weeks of bracing was recommended at 23 h per day, however given the rate of improvement and difficulty with compliance, the parents decided to cease bracing. At 13 months of age a trial of manual therapy commenced.

### Physical examination/diagnostic assessment

Initial examination at 13 months of age revealed a non-ambulatory female toddler demonstrating general low muscle tone. There was full head lag on Pull-to-Sit test, Ventral Suspension Test showed an inability to maintain the head at or above horizontal, and Vertical Suspension Test revealed poor upper body tone. Moro reflex was present with a symmetrical phase 1 and 2, however both Galant's Spinal and Perez reflex were absent. Muscle stretch reflexes were bilaterally absent for C5, C6, C7, L4, and S1 nerve roots.

Spinal passive range of motion assessment identified restriction in cervical lateral flexion, flexion, and rotation. Further restrictions in motion were palpated at the S2 sacral segment and coccyx. Allis test revealed a positive left Galeazzi sign. Thomas and Klisic's test were unremarkable. Hip abduction test was symmetrical but limited to 90° due to supine position on examination table. Unilateral straight leg abduction demonstrated asymmetry, with the right leg reaching 160° abduction, and the left limited to 80°.

Infant Neurological International Battery (INFANIB) assessment was performed at the second consultation assessing lower limb tone: Popliteal angle was greater than 170° (one stage above expected for age), Bilateral Leg Abduction was greater than 150°, Heel to Ear less than 10° (one stage above expected for age), and Foot Dorsiflexion of 0–10° bilaterally (three stages below expected for age).

### Therapeutic intervention

Treatment consisted of spinal manipulative therapy modified for patient age and size. A “Thuli”-branded portable drop piece (Thuli Tables Inc, Dodgeville, WI, USA) was used to assist manual therapy to spinal segments/vertebrae demonstrating articular restriction; C0/1, S2/3, and Coccyx 1/2.

Treatments were performed with a parent present after informed consent had been obtained. Primary outcomes of treatment were measured as parent reported and clinician observed improvements in gross motor development, normalization of muscle stretch reflexes, objective improvement in lower limb tone, and increased spinal range of motion. Secondary outcomes were aimed at improvements in hip range of motion and acetabular angle. Consent for the use of patient data for publication as a case study was obtained. There were no adverse events reported during or as a result of treatment.

### Follow-up and outcomes

Following the initial treatment (T1, day 0, 13M5D), examination showed improvements in lower limb muscle stretch reflexes, listed as +2 (L4) and +1 (S1). Lower limb tone on INFANIB remained unchanged. The parents observed that she was more active and cruising along furniture more frequently.

At T3 (day 14, 13M19D), parents reported that she had started walking one step independently. INFANIB tone screening showed mild improvements in all aspects of testing. Vertical suspension test shoulder girdle muscle tone was slightly decreased and Pull-to-Sit demonstrated mild lag.

At T5 (day 28), parents noted that she was walking a few steps independently between pieces of furniture, however a Trendelenburg-style limp was observed. Examination at this appointment revealed no changes when compared to initial hip examination, but a restriction in knee joint passive motion was detected and corrected utilizing the portable drop-piece. By T8 (day 63), parents reported independent and confident walking up to 12–14 steps without any apparent limp.

After the ninth treatment over a 12-week period (16M6D), a review radiograph was obtained by her orthopaedic specialist, with acetabular angles of 27° (right) and 29° (left) measured. Continuation of bracing was recommended by the orthopaedic specialist, but the parents chose not to comply.

On T10 (day 91, 16M7D), parents reported walking independently without limit. Examination revealed bilateral +3 muscle stretch reflex of L4 and S1 nerve root, and neutral but not flexed head position with Pull-to-Sit test. INFANIB lower limb screen revealed a trend toward normalization of appropriate tone for age, with hip abduction symmetrical and approximating 90° when tested unilaterally. Spinal and extremity range of motion assessment was full and symmetrical, with no indication of joint restriction or dysfunction present. At this point, the trial of treatment was considered complete, however parents opted to continue review appointments to ensure no regression occurred. A further three consultations occurred at monthly intervals, with manual therapy provided to cervical and sacral regions as indicated.

A second review radiograph was arranged by her orthopaedic specialist (18M30D) which demonstrated acetabular angles of 27° left and 26° right, with symmetrical curvature of both acetabular regions and no indication of hip dysplasia (Figure 1B).

## Case 2

### Patient information

The patient was a 30-month-old female born at 38 weeks *via* emergency C-section. Oligohydramniosis was detected on ultrasound at 37 + 5 weeks gestation. The mother was P1G1 and required IVF conception. There were substantial difficulties with breastfeeding resulting in breastfeeding cessation at 6 weeks of age. She was on a solids diet without indication of dysphagia.

The primary reason for seeking care was gross motor delay. Independent sitting was attained at 9 months of age, hands and knees crawling occurred from 11 months of age, and cruising along furniture from 13 months of age. Independent walking had not been achieved by 20 months of age, at which time bilateral casting followed by Ankle/Foot Orthoses (AFO) for Achilles Tendon contracture occurred over 6 months. Independent ambulation without AFO was attained at 28 months of age, however a waddling gait was observed. This raised suspicion of late-diagnosed DDH and an x-ray was performed at 30 months of age showing bilateral hip dysplasia (Figure 1C). A recommendation to commence Rhino bracing for 23 h per day was advised by an orthopaedic surgeon, however an option for non-bracing conservative management was sought by the parents prior to commencing bracing.

X-ray performed at 2 years, 6 months, and 3 days of age (2Y6M3D) demonstrated acetabular angles of 31.6° right, 32° left. Femoral neck-shaft angle was 162.3° (right) and 166.6° (left). Utilizing the International Hip Dysplasia Institute (IHDI) classification of hip dysplasia Case hips were borderline 2B bilaterally (25, 27).

### Physical examination/diagnostic assessment

Examination performed at 30 months of age (2Y6M7D) showed an ambulatory toddler walking with a pronounced waddling gait. A truncated neurological examination was performed due to lack of patient compliance. However, it revealed diminished (+2) L4 MSR bilaterally, with an absent right and strongly diminished (+1) left S1 MSR. Spinal passive range of motion assessment demonstrated restriction in bilateral cervical lateral flexion and rotation, with decreased motion palpated in sacral segments 2–4 and sacrococcygeal junction. Extremity examination showed bilateral restriction in ankle dorsiflexion. Hip assessment found no abnormality in the Telescoping test, Thomas test, or Allis test. Galeazzi sign was absent. Hip abduction testing in both straight-leg and knee flexed showed approximately 45° hip abduction on the left and 80° on the right.

### Therapeutic intervention

Treatment consisted of spinal manipulative therapy modified for patient age and size. A “Thuli”-branded portable drop piece was used to assist manual therapy to spinal segments/vertebrae demonstrating articular restriction; C0/1, C2/3, and S2–4.

All treatments were performed with a parent present after informed consent had been obtained. Primary outcomes of treatment were measured as clinician observed and parent reported improvements in gross motor development, normalisation of muscle stretch reflexes, and increased hip joint as well as spinal range of motion. Secondary outcomes were aimed at improvements in hip acetabular angle and femoral neck-shaft angle. Consent for the use of patient data for publication as a case study was obtained. There were no adverse events reported during or as a result of treatment.

### Follow-up and outcomes

At T3 (30M14D), parents reported an improvement with the waddling gait, with less truncal sway apparent. Hip assessment at this appointment showed no changes to hip abduction, however increased tone on Thomas test was felt bilaterally. Passive range of motion assessment demonstrated reduction in cervical lateral flexion and sacral flexion restriction.

At T4 (30M21D), parents reported an increase in walking pace, with less sway apparent. At T7 (31M26D), parents reported toe-out occurring in the mornings which appears to improve as the day progresses. At T8 (32M2D), parents reported that she was beginning to jump on a trampoline. At T10 (32M16D), she started running in forward locomotion as well as on the spot. Hip examination at this appointment demonstrated normal hip findings; 90° bilateral hip abduction on knee-flexed and straight-leg testing. Gait analysis demonstrated reduced truncal sway and improved foot positioning.

A review radiograph was performed at 32 months of age (2Y8M26D) demonstrating a 2-degree improvement of the right hip and a one-degree improvement of the left.

At T12 (33M20D), parents reported that she has been walking comfortably up stairs however one leg at a time. At this stage the initial trial of treatment concluded, however parents opted to continue treatment.

A second review radiograph at 35 months of age (2Y11M10D) showed reduction of acetabular angle to 28.8° right and 27.8° left (Figure 1D). The IDHI Classification of hip dysplasia showed an improvement from borderline Grade 2B to Grade 1B. Femoral neck shaft angle had reduced to 153.1° (right) and 151° (left).

## Discussion

This paper chronicles two paediatric cases with late diagnosed DDH demonstrating improvement in hip morphology after a trial period of manual therapy. In both cases, repeat x-ray after a 3-month trial of manual therapy demonstrated an improvement in acetabular angle and femoral head position based on IHDI classification, as well as femoral neck shaft angle in Case 2.

Case 1 was a non-ambulant toddler who presented at 13 months of age seeking chiropractic management involving manual therapy, following a three-month trial of bracing with limited success, rather than commence a further three-month trial of bracing as suggested by the orthopaedic surgeon. Pelvic x-ray demonstrated right acetabular angle of 33° (L) and 24° (R) consistent with left DDH. Case 2 was an ambulation-delayed pre-schooler who had recently finished a period of AFO for bilateral Achilles tendon contracture. Pelvic x-ray demonstrated an acetabular angle of 32° (L) and 31.6° (R) consistent with bilateral hip dysplasia. Bracing for 3 months was recommended, however, her parents opted to trial non-bracing conservative management involving manual therapy.

**Figure 2:**
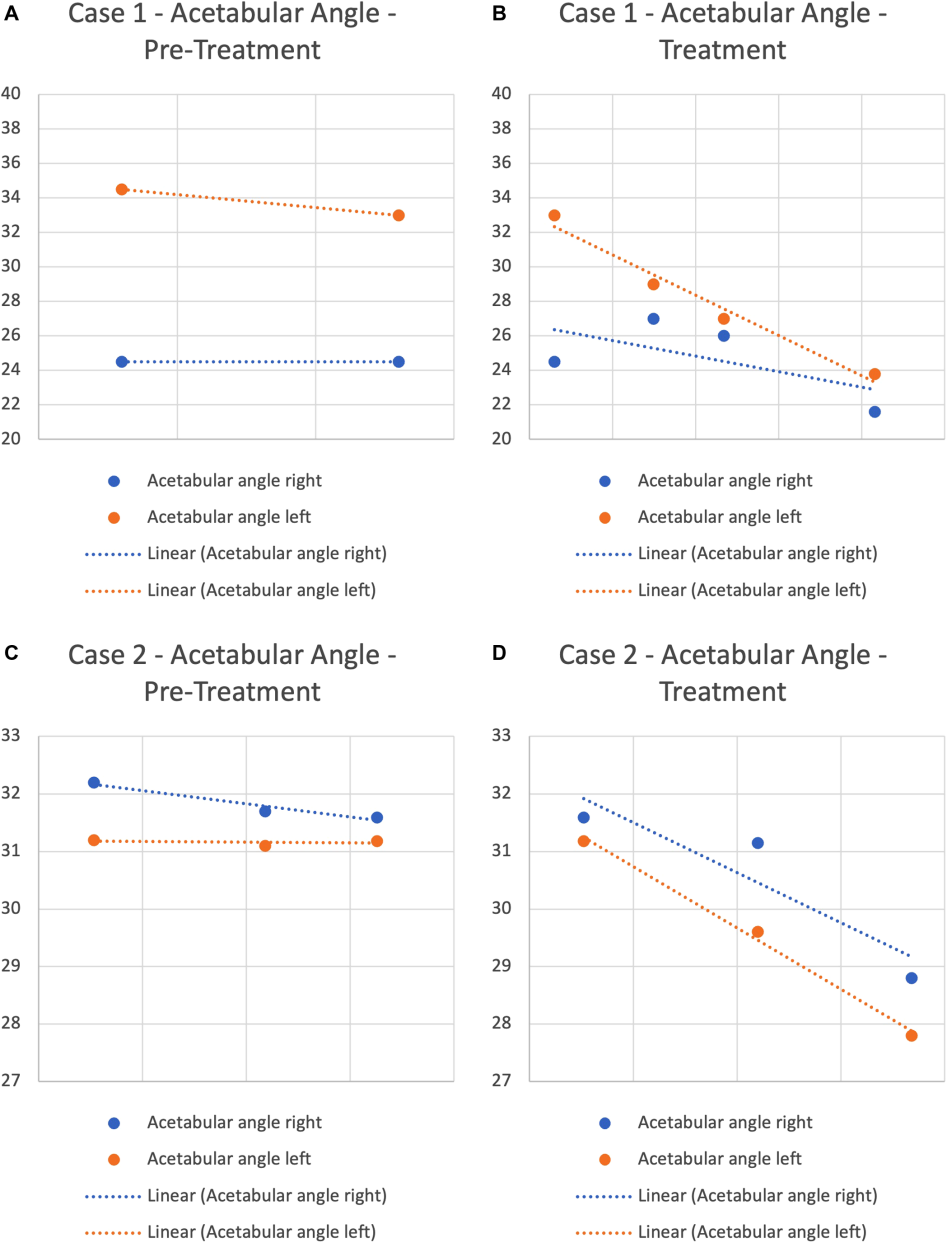
Acetabular angle progression pre-treatment and with treatment.

In Case 1, left acetabular angle reduced by 4° from 33° to 29° and a normalisation in acetabular shape was observed during a trial of manual therapy after a 6-month period (Figures 2A,B). While appreciating that association does not imply causation, given the extent and rate (4° over 6 months), the onset of improvement subsequent to initiation of manual therapy after a three-month period of bracing which did not substantially impact acetabular angles may be considered as having been a contributing factor to this change. The further 2-degree improvement of the left acetabular angle from 29° to 27° occurred over the following three months without use of a brace and may be due to ongoing improved mechano-transduction driven skeletal changes.

In Case 1, we observed gross motor developmental changes, normalisation of muscle stretch reflexes, and mild improvements in muscle tone. The toddler was walking independently with a Trendelenburg gait within 4 treatments, and confidently with no limp within 7 treatments. Treatment was manual therapy targeted to upper cervical and/or sacral spine.

With Case 1, as an infant with several risk factors and mild dysplasia evident on ultrasound monitoring did not occur between 3.5 and 9 months of age as recommended by guidelines. Regardless of the presence or suspicion of DDH in infants, best practice recommends surveillance screening until walking age, with timeframes outlined in at least 4 Australian guidelines in accordance with the American Academy of Paediatrics (3, 28–32). The Australian College of Chiropractic Paediatrics recommends hip joint screening beyond 4 months of age; at 6, 8 and 10 months of age (33).

**Figure 3:**
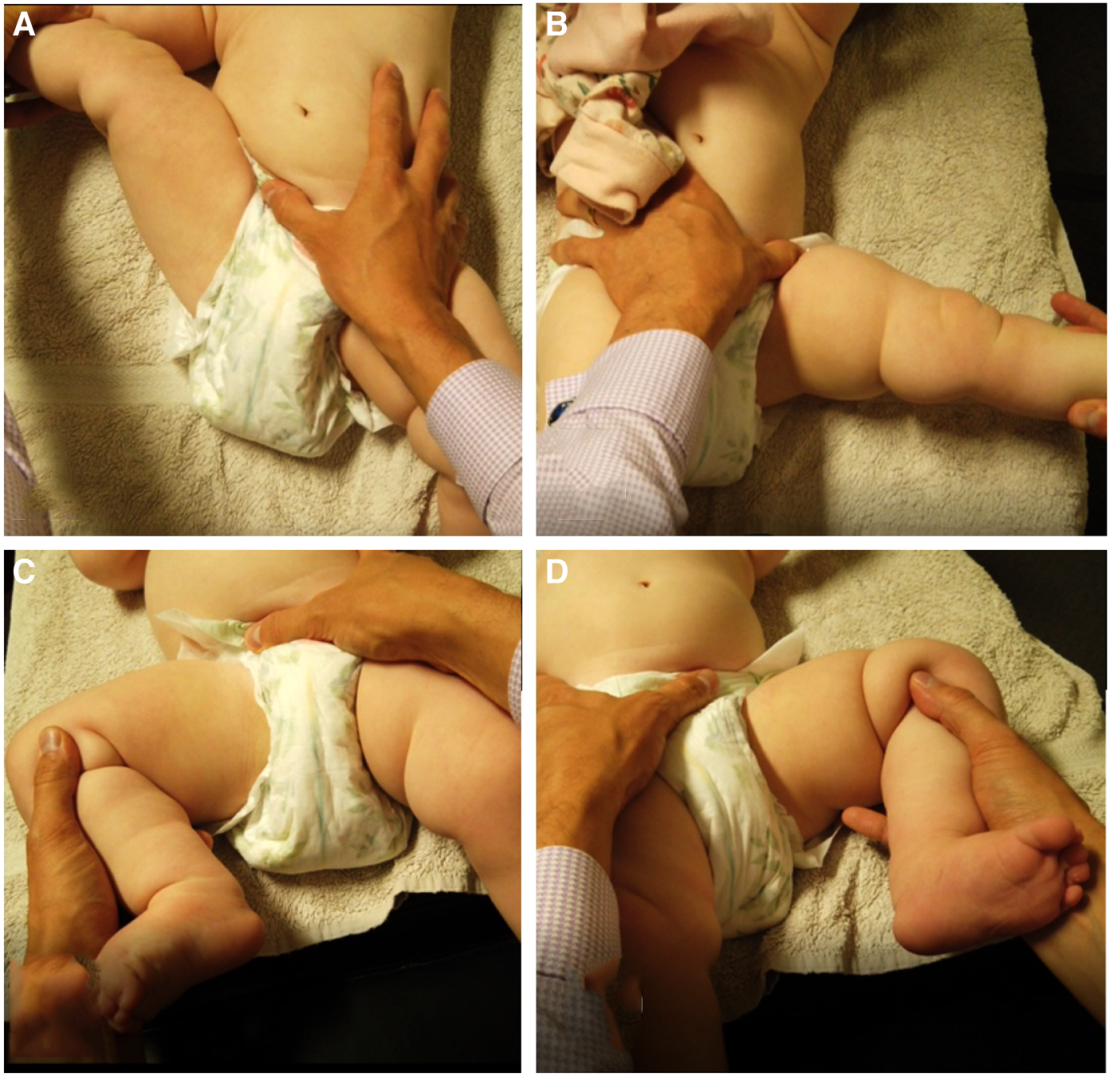
“Unilateral Straight Leg Abduction Test”, (**A**) unilateral straight leg abduction right demonstrating >90° abduction, (**B**) unilateral straight Leg abduction left demonstrating <90° abduction, (**C**) normal right hip abduction test, (**D**) marginally reduced left Hip abduction test, within normal parameters of <20° difference. Thigh crease asymmetry noted on the left.

Case 1 demonstrated a conflict between standard hip examination and x-ray results. Standard hip abduction tests when performed at 90° abduction yielded results within normal limits, however upon performing a modified form of the leg abduction test (leg straight or fully extended at hip and knee joints) from the INFANIB, as recommended and taught by one of the authors (BK), an asymmetry in hip abduction was observed (Figure 3). Performed in a unilateral manner as opposed to bilateral, this test was able to demonstrate a substantial difference in leg abduction, and while it may not be a validated hip test, it was able to demonstrate a clear difference in symmetry. Further investigation to the validity of this test, which we have coined the “Unilateral Straight Leg Abduction Test”, may be warranted.

In Case 2, x-ray performed after 3 months of manual therapy showed a small change in acetabular angle, with the left reducing by 2°(29.6°) and the right by one degree (31.1°). However, H-point position had improved, with the IHDI hip severity reducing from borderline Grade 2 to Grade 1 bilaterally (Figures 2C,D). A second x-ray 2.5 months later demonstrated continued change; the left acetabular angle reducing about 2° to 27.8°, and the right 2.3° to 28.8° (Figure 1D).

**Figure 4:**
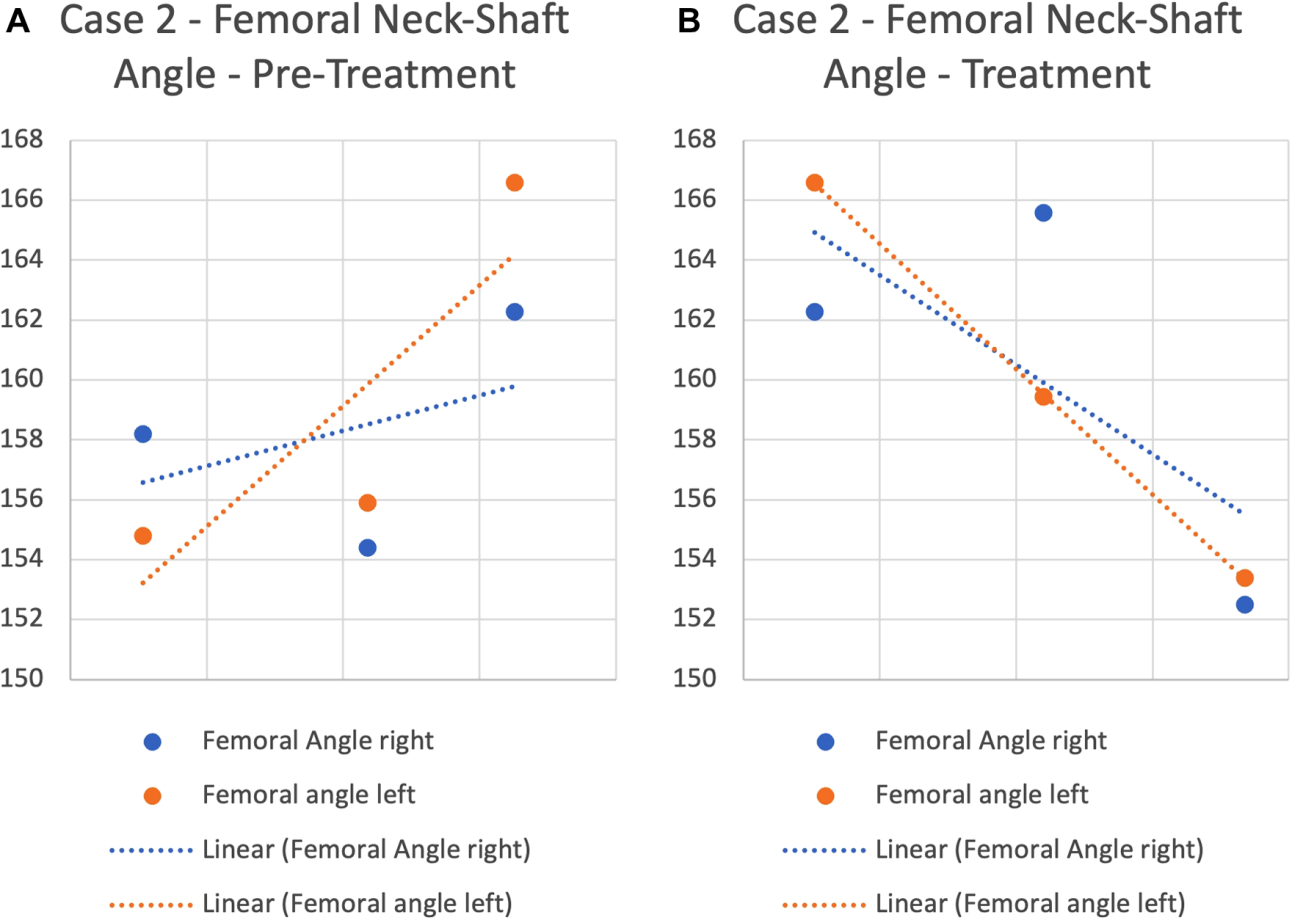
Case 2 femoral neck-shaft angle pre-treatment and after treatment commenced.

In this timeframe, the femoral neck angle reduced from its initial angle of 162.3° (right) and 166.6° (left) to 153.1° (right) and 151° on the left (Figures 4A,B). At 3 years of age, the femoral neck-shaft angle is expected to reduce from 150° in neonate age to 145° (34). Greater than 150° is consistent with DDH (35).

In Case 2, we saw catch up of gross motor developmental milestones, with each milestone occurring closer to the expected age of development; walking at 28 months of age, a 13–16-month delay; running at 32 months of age, an 8–14-month delay; ascending and descending stairs at 33 months of age, a 3–8-month delay. Postural gait analysis indicated an improvement in walking posture, Trendelenburg gait and limp.

Spontaneous resolution of DDH does occur, with Type 2a+ normalising in 83%–98% of cases typically occurring within the first three months of age (36). Spontaneous resolution beyond six months of age without intervention is unlikely, and often requires invasive intervention for normalisation as the child ages (18). Acetabular remodelling is regarded as a slow process; literature is outdated and inconsistent in determining rates of improvement with one author stating that it is unlikely to occur after 18 months of age (37) and others noting it can take up to eight years (38, 39). Understanding the slow rate of resolution involved in children over 6 months old, and the apparent lack of improvement with bracing after this age, it is difficult for practitioners to provide suitable management options for children older than 6 months of age with hip dysplasia.

The unexpected and rapid improvements recorded in these two cases undergoing manual therapy raises the question of the biological mechanism explaining these changes. The process of mechanotransduction, which is responsible for development of joints including the acetabulum, involves transmission of force along a particular plane resulting in changes to cartilage and bone (40). Skeletal osseous structures are well known to respond to changes in applied forces. In the reported cases, improvements in muscle tone and neurodevelopment such as gross motor function were apparent while receiving manual therapy designed to improve sacral and cervical spine joint function. With the hip enlocated, the altered transmission of force through the hip articulation as a result of improved muscle tone and improved weight bearing drove mechanotransductive induced changes to the structure and shape of the acetabulum and femoral neck (2). Improvement with hip dysplasia associated with manual therapy treatment has not previously been published with a search of Medline, CINAHL, and Index to Chiropractic Literature utilising search terms “toddler”, “manual therapy”, and “hip dysplasia” yielding no results.

Changes in somatosensory processing, tone and cortical muscle drive have been reported after spinal and extremity joint manipulative therapy in numerous studies (41–45). Haavik (2016) and Navid (2022) both report increases in lower limb strength after spinal manipulation attributed to increased descending cortical drive (43, 45). Haavik et al. (2021) further details neurological pathways that explain both motor and developmental progress observed after spinal and extremity joint manipulative therapy (46). While these data are reported on adult populations, the mechanism of action would be expected to be similar with children over 12 months of age. In the two reported cases, changes to motor drive and tone coupled with development of locomotion provides a mechanism which promotes mechanotransductive changes with the acetabulum and femur.

The rate of normalisation reported in both Case 1 and Case 2 is not consistent with the natural course of DDH. It seems possible the trial of manual therapy may have contributed to the positive outcome, and no detrimental effect of the treatment on either child was reported. Manual therapists, particularly those who have received further education and training in the presentation and management of common paediatric conditions, are well placed to understand and address the neuromusculoskeletal drivers and limitations to hip structure and function.

There are limitations extrapolating data from case studies. We are unable to exclude initial bracing performed as being a factor for hip initiating normalisation in Case 1, nor spontaneous resolution however unlikely. There is no data to aid in determining ideal window of treatment; whether these cases have responded more favourably if treatment had commenced prior to 12 months old and in ambulatory individuals is uncertain. We do have a biologically plausible mechanism by which mechanotransduction may be responsible for the changes observed. Future prospective trials utilising manual therapy as an adjunctive therapy to dynamic bracing in infants and toddlers older than 6 months of age may be warranted.

Case studies such as this are valuable to highlight the role manual therapy may play as a conjunctive therapy to dynamic bracing in infants and toddlers older than 6 months of age. Given as an option without clear, evidence-based support, parents of Case 1 provided insightful feedback regarding this outcome: “Our experience with [manual therapy] for DDH has been extremely positive. We arrived as parents wanting to provide the best opportunities and outcomes for our child. Neither of us had experienced [manual therapy] before and were unsure if it would help but were willing to try anything. We were put at ease throughout the process, felt included in our daughters care and could see immediate differences after [manual therapy]. Our child would have assessments on her reflexes and her tone would be checked. We have had huge successes with [manual therapy] and are so grateful that we reached out to try something that we were not familiar with.”

Only through a collection of experiences can we develop hypotheses from which to design further quality research into the role manual therapy may play in the management of late diagnosed DDH.

## Conclusion

After a trial of manual therapy, morphological changes suggestive of improvement of acetabular and femoral neck shaft angle were evident in two cases of late-diagnosed DDH. Given the paucity of research for management of non-surgical DDH cases and the limited rate of success with current conservative management options, a trial of manual therapy in conjunction to bracing may be of benefit. Further research is required.

## Data Availability

The original contributions presented in the study are included in the article/Supplementary Material, further inquiries can be directed to the corresponding author/s.
